# Supplementary material for: Human colon stem cells are the predominant epithelial responders to bacterial antigens
Source: Front Immunol. 2025 Oct 15;16:1677943. doi: 10.3389/fimmu.2025.1677943 (PMC12568582; doi:10.3389/fimmu.2025.1677943)

Supplementary figure 1

A

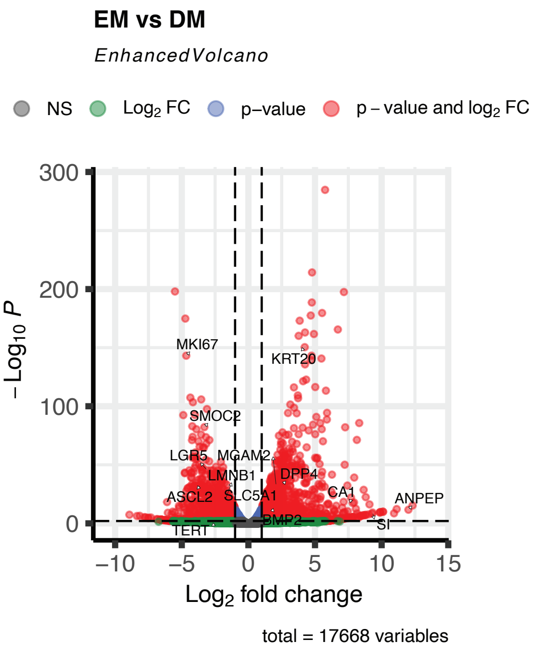

B

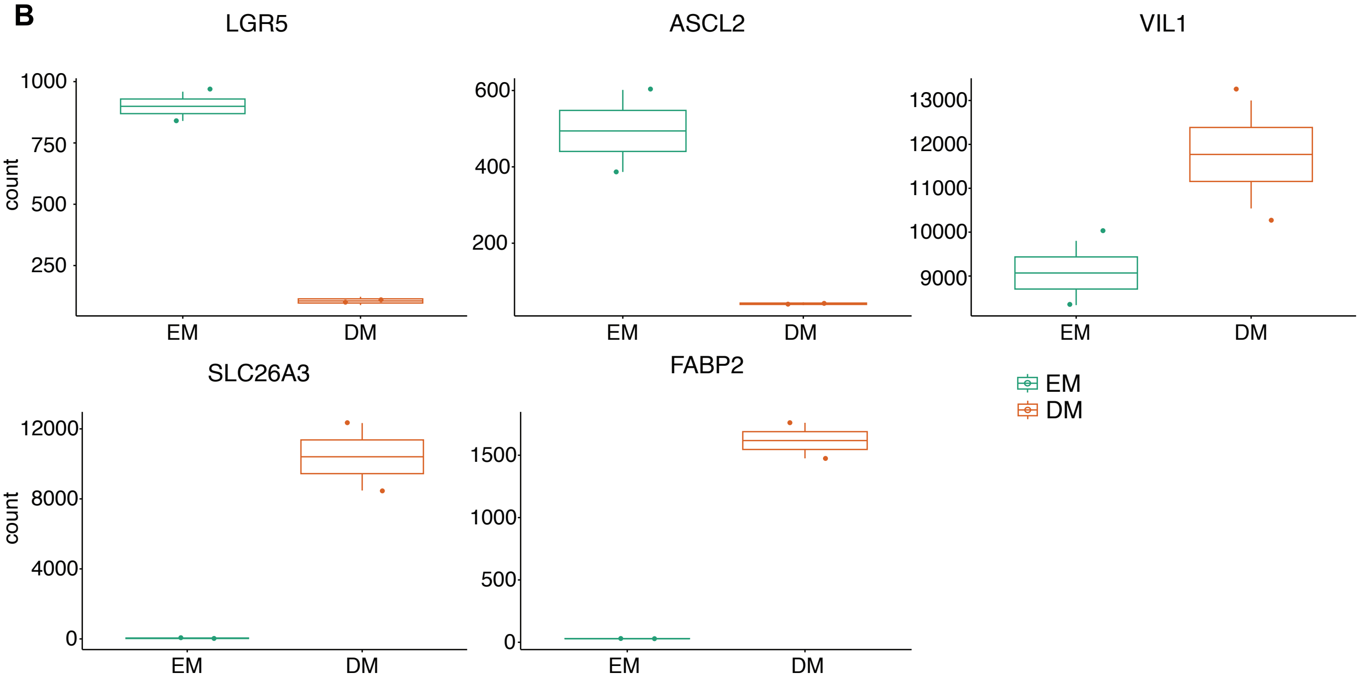

Supplementary figure 2

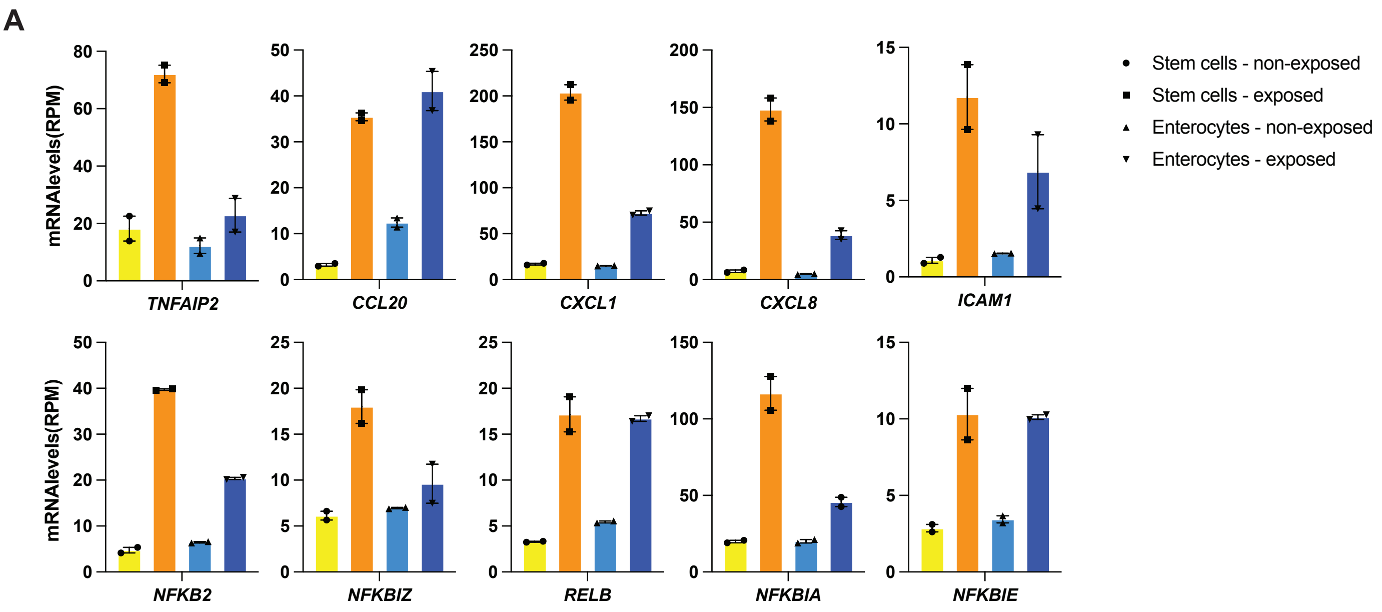

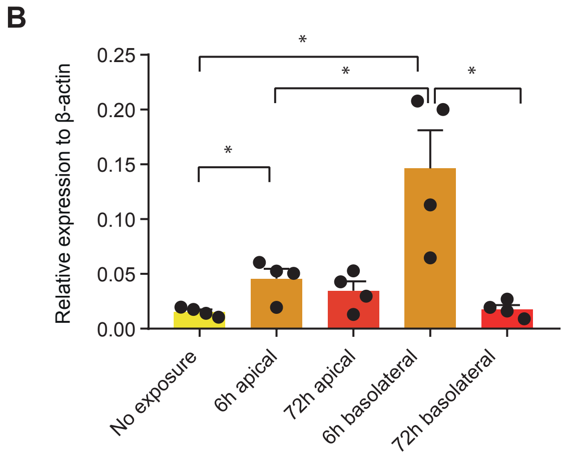

Supplementary figure 3

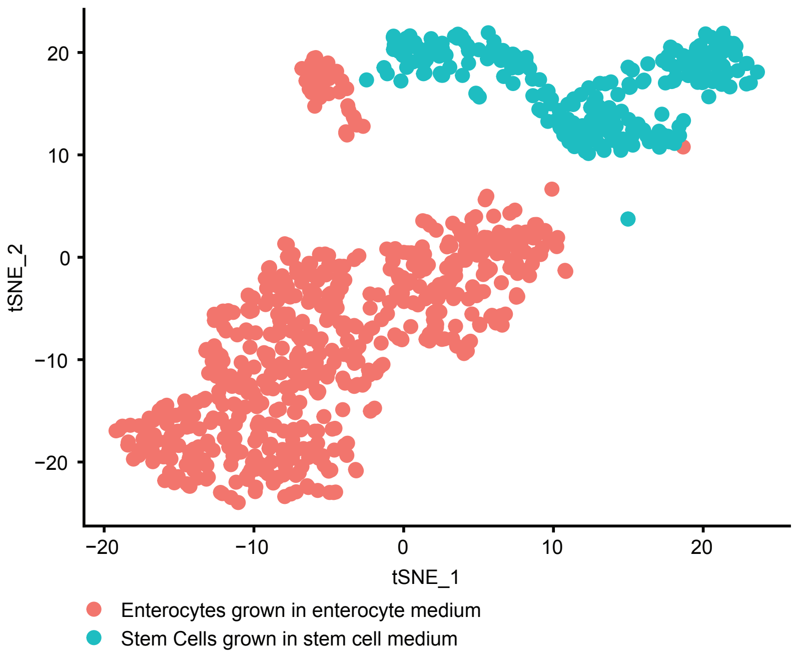

Supplementary figure 4

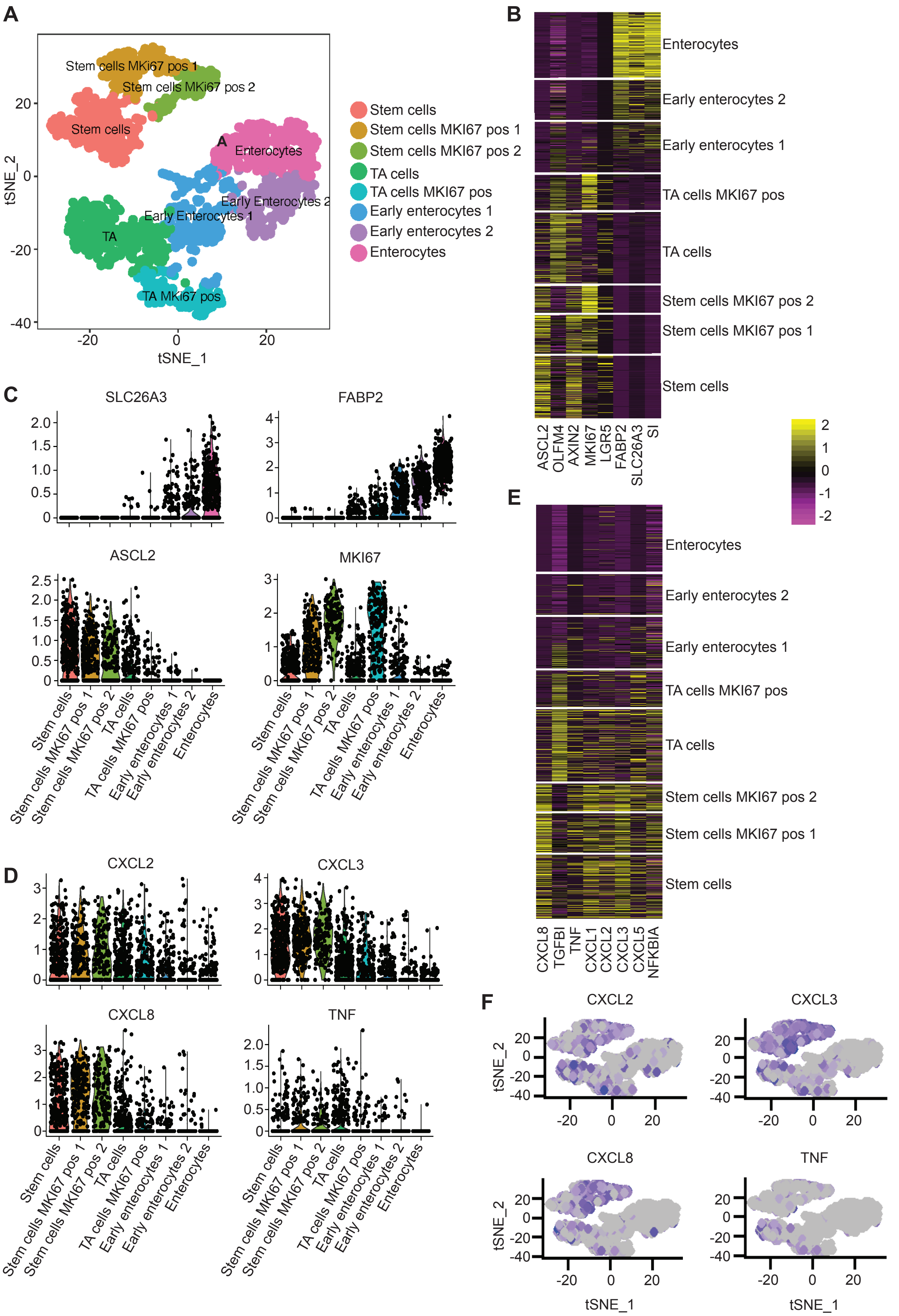

Supplementary figure 5

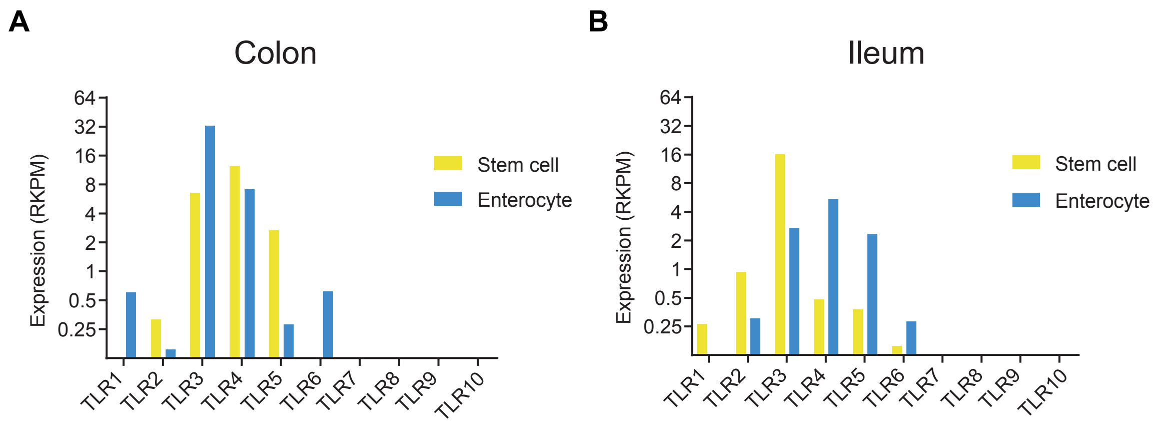

Supplementary figure 6

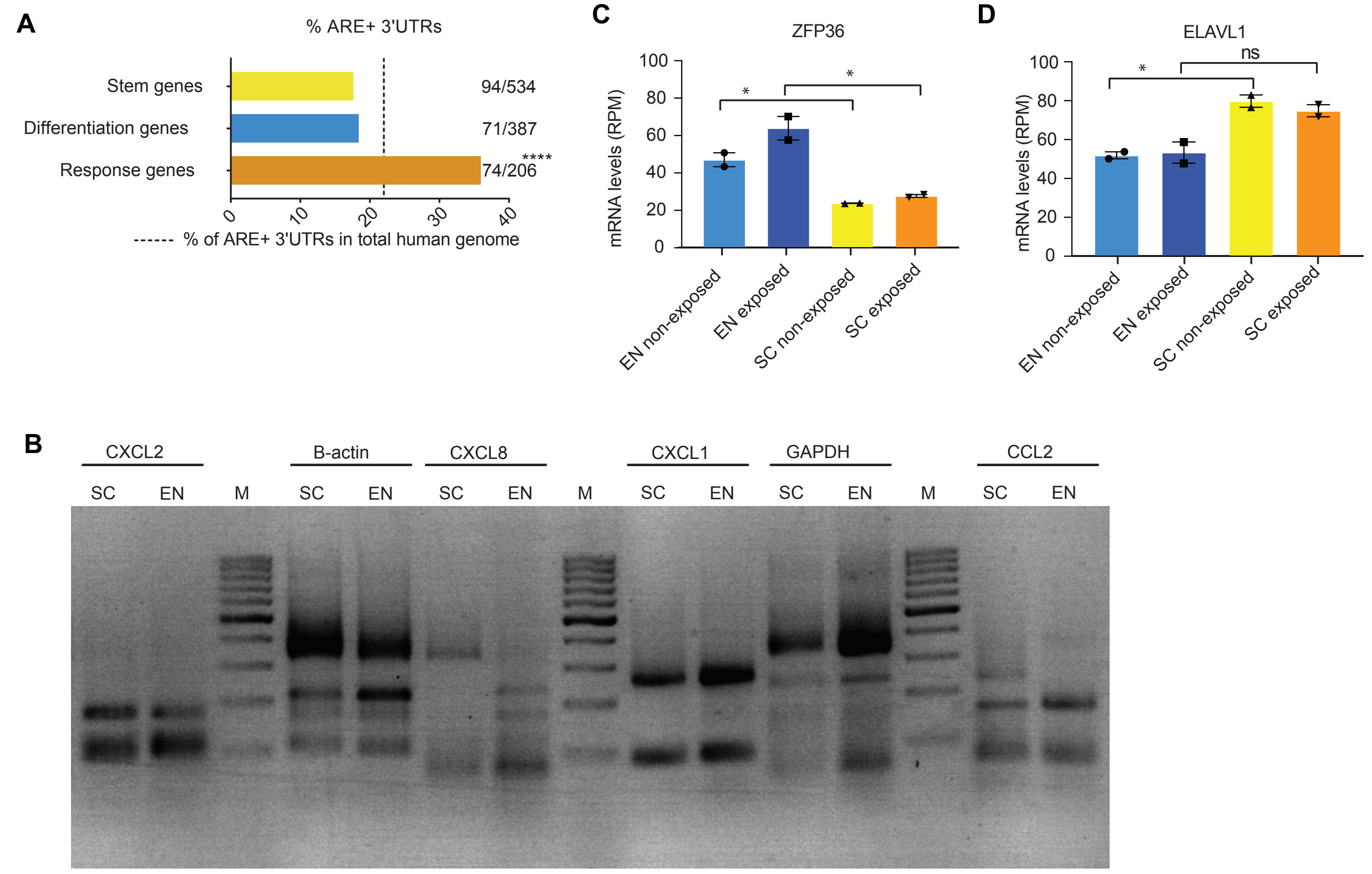

Supplementary figure 7

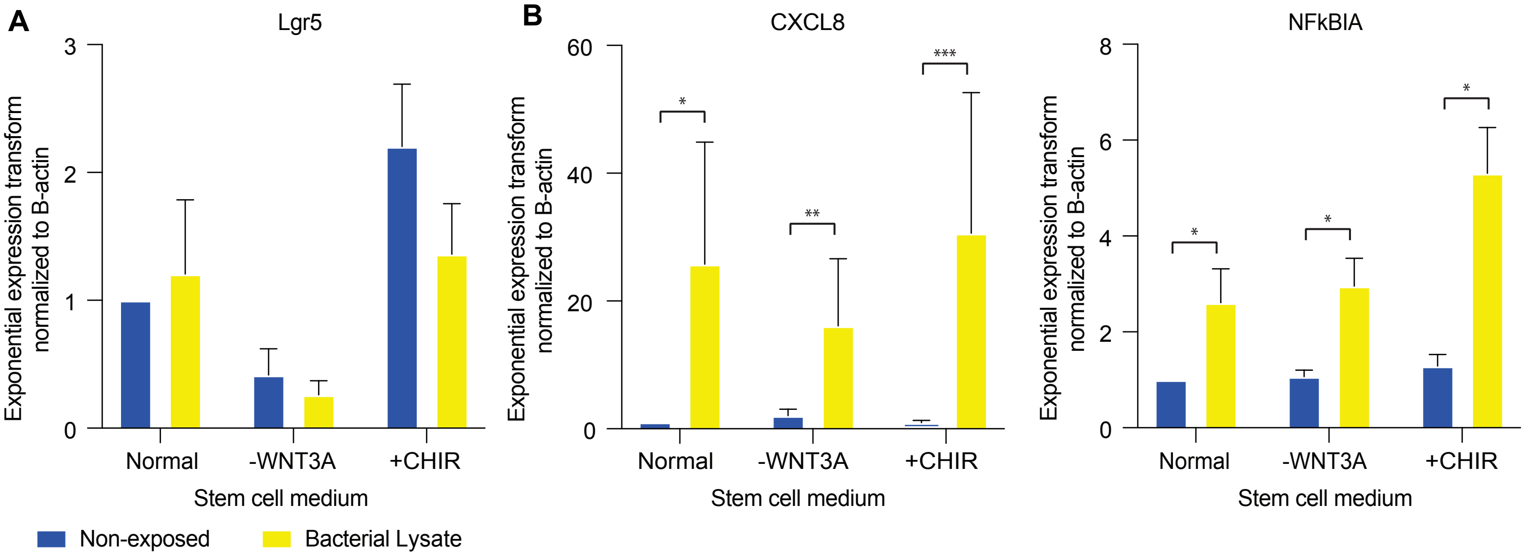

Supplement: Supplementary Figure 1 — (A) Volcano plot of bulk RNAseq of colon enterocytes-enriched organoids 0h versus colon intestinal stem cells-enriched organoids 0h (n=2) (B) Boxplot of intestinal stem cell markers (LGR5, ASCL2) and differentiation markers (VIL1, SLC26A3, and FABP2) in enterocyte- and intestinal stem cell-enriched colon organoids. (n=2). [file DataSheet1.pdf]
